# Meta-analysis of human genome-microbiome association studies: the MiBioGen consortium initiative

**DOI:** 10.1186/s40168-018-0479-3

**Published:** 2018-06-08

**Authors:** Jun Wang, Alexander Kurilshikov, Djawad Radjabzadeh, Williams Turpin, Kenneth Croitoru, Marc Jan Bonder, Matthew A. Jackson, Carolina Medina-Gomez, Fabian Frost, Georg Homuth, Malte Rühlemann, David Hughes, Han-na Kim, MiBioGen Consortium Initiative, Tarun Ahluwalia, Elad Barkan, Larbi Bedrani, Jordana Bell, Hans Bisgaard, Michael Boehnke, Marc Jan Bonder, Klaus Bønnelykke, Dorret I. Boomsma, Kenneth Croitoru, Gareth E. Davies, Eco de Geus, Frauke Degenhardt, Mauro D’Amato, Erik A. Ehli, Osvaldo Espin-Garcia, Casey T. Finnicum, Myriam Fornage, Andre Franke, Lude Franke, Fabian Frost, Jingyuan Fu, Femke-A. Heinsen, Georg Homuth, David Hughes, Richard IJzerman, Matthew A. Jackson, Leon Eyrich Jessen, Daisy Jonkers, Tim Kacprowski, Han-Na Kim, Hyung-Lae Kim, Robert Kraaij, Alex Kurilshikov, Markku Laakso, Lenore Launer, Markus M. Lerch, Kreete Lüll, Aldons J. Lusis, Massimo Mangino, Julia Mayerle, Hamdi Mbarek, Maria Carolina Medina, Katie Meyer, Karen L. Mohlke, Elin Org, Andrew Paterson, Haydeh Payami, Djawad Radjabzadeh, Jeroen Raes, Daphna Rothschild, Malte Rühlemann, Serena Sanna, Eran Segal, Shiraz Shah, Michelle Smith, Tim Spector, Claire Steves, Jakob Stokholm, Joanna W. Szopinska, Jonathan Thorsen, Nicolas Timpson, Williams Turpin, André G. Uitterlinden, Alejandro Arias Vasquez, Henry Völzke, Urmo Vosa, Zachary Wallen, Jun Wang, Frank Ulrich Weiss, Omer Weissbrod, Cisca Wijmenga, Gonneke Willemsen, Wei Xu, Yeojun Yun, Alexandra Zhernakova, Tim D. Spector, Jordana T. Bell, Claire J. Steves, Nicolas Timpson, Andre Franke, Cisca Wijmenga, Katie Meyer, Tim Kacprowski, Lude Franke, Andrew D. Paterson, Jeroen Raes, Robert Kraaij, Alexandra Zhernakova

**Affiliations:** 1 CAS Key Laboratory for Pathogenic Microbiology and Immunology, Institute of Microbiology, Chinese Academy of Sciences, Beijing, China; 2 Department of Microbiology and Immunology, Rega Institute. KU Leuven – University of Leuven, Leuven, Belgium; 3 VIB Center for Microbiology, Leuven, Belgium; 4 Department of Genetics, University of Groningen, University Medical Center Groningen, Groningen, The Netherlands; 5 Department of Internal Medicine, Erasmus Medical Center, Rotterdam, The Netherlands; 6 Division of Gastroenterology, Department of Medicine, University of Toronto, Toronto, Ontario Canada; 7 Zane Cohen Centre for Digestive Diseases, Mount Sinai Hospital, Toronto, Ontario Canada; 8 European Molecular Biology Laboratory, European Bioinformatics Institute, Hinxton, UK; 9 Department of Twin Research and Genetic Epidemiology, King’s College London, London, UK; 10 The Generation R Study Group, Erasmus MC, 3000 CA Rotterdam, The Netherlands; 11 Department of Epidemiology, Erasmus MC, 3000 CA Rotterdam, The Netherlands; 12 Department of Medicine A, University Medicine Greifswald, Greifswald, Germany; 13 Department of Functional Genomics, Interfaculty Institute for Genetics and Functional Genomics, University Medicine Greifswald, Greifswald, Germany; 14 Institute of Clinical Molecular Biology, Christian Albrechts University of Kiel, Kiel, Germany; 15 MRC Integrative Epidemiology Unit at University of Bristol, Bristol, UK; 16 Population Health Sciences, Bristol Medical School, University of Bristol, Bristol, UK; 17 Department of Biochemistry, School of Medicine, Ewha Womans University, Seoul, South Korea; 18 Department of Nutrition, Nutrition Research Institute, University of North Carolina at Chapel Hill, Kannapolis, NC USA; 19 Division of Biostatistics, Dalla Lana School of Public Health, University of Toronto, Toronto, Ontario Canada; 20 Division of Epidemiology, Dalla Lana School of Public Health, University of Toronto, Toronto, Ontario Canada; 21 Genetics and Genome Biology, The Hospital for Sick Children Research Institute, The Hospital for Sick Children, Toronto, Ontario Canada

**Keywords:** Gut microbiome, Genome-wide association studies (GWAS), Meta-analysis

## Abstract

**Background:**

In recent years, human microbiota, especially gut microbiota, have emerged as an important yet complex trait influencing human metabolism, immunology, and diseases. Many studies are investigating the forces underlying the observed variation, including the human genetic variants that shape human microbiota. Several preliminary genome-wide association studies (GWAS) have been completed, but more are necessary to achieve a fuller picture.

**Results:**

Here, we announce the MiBioGen consortium initiative, which has assembled 18 population-level cohorts and some 19,000 participants. Its aim is to generate new knowledge for the rapidly developing field of microbiota research. Each cohort has surveyed the gut microbiome via 16S rRNA sequencing and genotyped their participants with full-genome SNP arrays. We have standardized the analytical pipelines for both the microbiota phenotypes and genotypes, and all the data have been processed using identical approaches. Our analysis of microbiome composition shows that we can reduce the potential artifacts introduced by technical differences in generating microbiota data. We are now in the process of benchmarking the association tests and performing meta-analyses of genome-wide associations. All pipeline and summary statistics results will be shared using public data repositories.

**Conclusion:**

We present the largest consortium to date devoted to microbiota-GWAS. We have adapted our analytical pipelines to suit multi-cohort analyses and expect to gain insight into host-microbiota cross-talk at the genome-wide level. And, as an open consortium, we invite more cohorts to join us (by contacting one of the corresponding authors) and to follow the analytical pipeline we have developed.

**Electronic supplementary material:**

The online version of this article (10.1186/s40168-018-0479-3) contains supplementary material, which is available to authorized users.

## Background

Our understanding of the microbial communities populating the human body (human microbiota) has progressed tremendously in recent years, catalyzed by the use of next-generation sequencing techniques that overcome the limitations of anaerobic cultivation [1]. Much effort has been devoted to understanding the taxonomic and functional diversity of the microbiota and their encoded collective gene pool, the microbiome, with most research activity focusing on the microbes in our gastrointestinal tract [2, 3]. Much of the research has centered on elucidating links between microbes and various diseases [4], for instance, obesity, inflammatory bowel disease, and diabetes. This has including several studies that went beyond association to demonstrate causal roles of the gut microbiome in disease development.

More knowledge of the microbial ecosystem and the role of different factors in its structure is an essential path leading to more understanding of human biology [5]. Cross-sectional studies carried out in several population-based cohorts have identified the major environmental factors (nutrition, medication, and diet) influencing the composition and functional capacities of the human microbiome [6, 7]. Yet these studies also showed that a large proportion of microbial diversity remained unexplained after considering the environmental influences, thereby raising questions on the role of host genetics.

Given the complex interplay between the microbiome and host physiology, a certain percentage of host genetics, as well as genetic interactions with environmental factors, is expected to shape the composition of the microbial community [8]. Proof-of-principle genome-wide screens (e.g., quantitative trait loci (QTL) studies) have been carried out in model organisms like mouse [9], while the majority of published studies on humans have used a candidate gene approach to cope with sample size limitations. Recently, analyses of twin cohorts have demonstrated a genetic contribution to variation in the relative proportions of specific members of microbiota [10], for example, investigations in 1126 twins identified associations to 28 loci, including genetic variants in *LCT* [11].

**Fig. 1:**
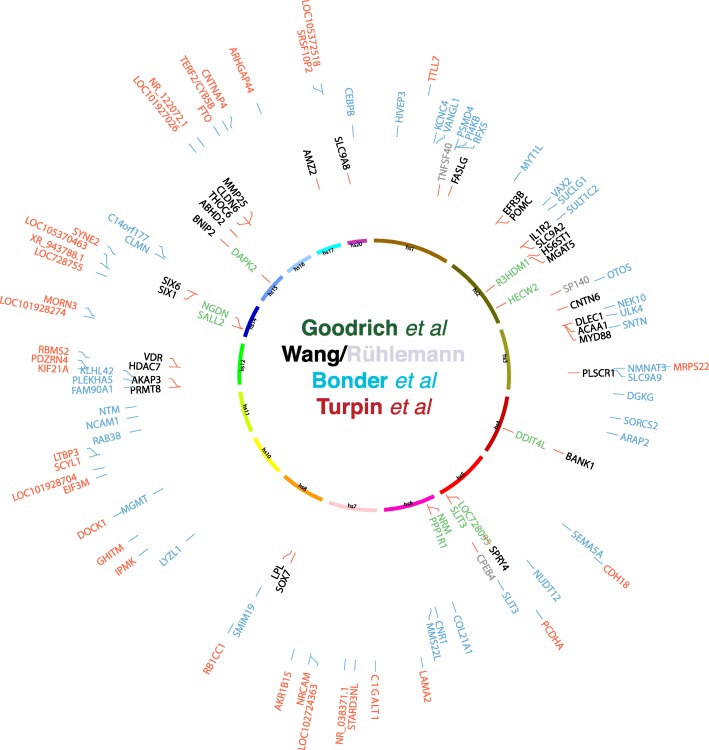
Overview of genome-wide significant loci discovered in four recent GWAS studies [9–12]. For simplicity, only the regions harboring a coding gene are shown, and for Wang et al. [14], the list was further refined to genes implicated in previous mouse QTL studies and to additional loci identified by an improved method (shown in gray, Rühlemann et al. Gut microbes, 2017). So far, the only overlap found in the three studies is the *SLIT3* locus, although two studies reported two SNPs not in linkage disequilibrium. The *LCT* locus was not significant in the initial analysis using an additive model, but analyzing functional SNPs in the recessive model identified a significant association for *LCT* in the Dutch cohort [15]

Bonder et al., Turpin et al.*,* and Wang et al. then simultaneously reported GWAS results from three independent cohorts, each revealing glimpses into the genetic landscape underlying the gut microbiota structure [12–14]. Together, these GWAS have identified some 100 genome-wide significant loci associated with community structure, taxon abundance, and gut microbiome biodiversity. However, similar to initial GWAS efforts in many other complex traits, there was little overlap seen in the three sets of summary statistics (Fig. 1). *SLIT3* was the only gene to pass a standard genome-wide significance threshold of 5 × 10^−8^ in the TwinsUK and Bonder et al. studies [11, 12], but the two reported single nucleotide polymorphisms (SNPs) within this gene are not proxies of each other, nor do they correlate to the same bacteria or pathway. Despite little overlap in the associated genetic variants, which were limited to the *LCT* locus, associations to various C-type lectin genes were observed by both Bonder et al. and Wang et al. [11, 12, 14]. These discordances emphasize the need to increase the number of samples in the discovery setting to improve statistical power and to reduce the probability of false-positive associations. Cross-multi-cohort analysis will also overcome limitations imposed by population stratification as well as technical artifacts, including the differences in model choice [15].

We have therefore established the MiBioGen consortium to study the influence of human genetics on gut microbiota. This collaborative effort currently comprises 18 cohorts worldwide and new members will join us after completing their data collection. We aim to develop a uniform pipeline to allow maximum harmonization across the microbiome data and to use GWAS meta-analyses to provide a fuller picture of human gene-microbiome associations. Furthermore, since all the cohorts have been well phenotyped, their data will aid future investigations into other research questions.

### MiBioGen initiative and cohort descriptions

Most of the 18 studies participating in the consortium are prospective cohort studies in countries in Europe, Asia, and North America (Table 1). Besides genetics and microbiome data, the cohorts have also been deeply phenotyped, covering multiple individual outcomes (e.g., anthropometric, metabolic, disease-related). These cohorts also incorporate a wide age spectrum, including both children and adults. The number of individuals per cohort study ranges from 139 to 2482, with a total of 19,790 individuals (18,965 after quality control (QC)). In terms of both sample size and geographic distribution, the MiBioGen consortium is, to our knowledge, the most comprehensive effort for investigating host-genetics-versus-microbiome-associations on a population scale.

**Table 1:** Information on the 18 cohorts participating in the MiBioGen consortium to date

Cohort name	Population (ethnicity)	16S domain	Genotyping platforms used	Sample size (after QC)	Description
BSPSPC	Germany (Caucasian)	V1-V2	Illumina 550K, Immunochip, Metabochip, Affymetrix 6.0, Axiom	912	Representative of population
CARDIA	USA (Caucasian and African-American)	V3-V4	Illumina Exome, Affymetrix 6.0	282	Representative of population
NeuroIMAGE + COMPULS	Netherlands (Caucasian)	V1-V2	PsychChip (Broad Institute, Boston, USA)	153	Healthy group + ADHD group
COPSAC	Denmark (Caucasian)	V4	Illumina OmniExpressExome	424	Children (unselected)
FGFP	Belgium (Caucasian)	V4	Illumina OmniExpressExome	2482	Representative of population
FoCus	Germany (Caucasian)	V1-V2	Illumina Immunochip, Exome	1555	Representative of population + obese sub-cohort
GEM	Canada, USA, Israel (Caucasian, Israeli)	V4	Illumina HumanCoreExome, Immunochip	1543	Healthy individuals
Generation R	Netherlands (multi-ethnic)	V3-V4	Illumina 610 k	2111	Representative of population
KSCS	South Korea (Eastern Asian)	V3-V4	Illumina HumanCore BeadChips 12v	833	Representative of population
LLD	Netherlands (Caucasian)	V4	Illumina Immunochip, Cytochip	1089	Representative of population
METSIM	Finland (Caucasian)	V4	Illumina OmniExpressExome	531	Representative of population
MIBS	Netherlands (Caucasian)	V4	Illumina OmniExpressExome	111	Healthy volunteers
PNP	Israel (Israeli)	V3-V4	Illumina Metabochip	1066	Healthy volunteers
Rotterdam Study	Netherlands (Caucasian)	V3-V4	Illumina 550k	1427	Representative of population
SHIP	Germany (Caucasian)	V1-V2	Affymetrix 6.0, Illumina OmniExpressExome, Exomechip	1904	Representative of population
TwinsUK	UK (Caucasian)	V4	HumanHap300, Hap610Q, 1M-Duo, 1.2M-Duo	1793	Twins
NTR	Netherlands (Caucasian)	V4	Affymetrix 6.0	499	Twins
PopCol	Sweden	V1-V2	Illumina MiSeq	250	Representative of population
Total				18,965	

As we have multiple phenotypes in addition to microbiome and host genotypes available, we can assess the putative effect of the gut microbiome on human health. Several of the cohorts were set up to investigate certain phenotypes and/or diseases, for instance, GEM (healthy relatives of patients with Crohn’s disease) [13], or FoCus (a nutritional intervention study) [14]. As a basis for epidemiological studies, various metadata were collected by the different cohorts including anthropometric measures, blood chemistry, dietary pattern, intestinal permeability, and lifestyle. These factors have been shown to influence microbiota composition [6, 7, 14]. All these metadata and phenotypes provide opportunities for assessing the biological significance of gene-microbiome associations, and for gaining insights into gene-environment interactions and the interaction between host genotype–microbiome–diseases.

## Methods

To provide a platform for robust and reliable results and also to simplify study participation in MiBioGen, we have standardized all the procedures and protocols that participating cohorts need to follow. The MiBioGen data processing pipeline comprises four steps: (1) microbiome data processing, (2) genotype data processing, (3) genome-wide association analyses, and (4) meta-analyses.

### Microbiome data processing

The microbiome data included in our consortium was mainly generated using an Illumina sequencing platform (MiSeq or HiSeq). The most frequently sequenced hyper-variable region of the 16S rRNA gene was V4 (eight cohorts, *n* = 8472), although five cohorts sequenced the V3-V4 region (*n* = 5719), and another four sequenced the V1-V2 region (*n* = 4774). We assessed the compatibility of the datasets obtained from sequencing different regions by comparing technical replicates of ten samples (three replicates each) generated from different hyper-variable regions. This analysis showed that the influence of technical differences in microbiome profiles is less than the inter-individual differences (Additional file 1). Nevertheless, including different hyper-variable regions requires compatible methods of 16S rRNA gene-amplicon data processing, and it is no longer feasible to use “open” (de novo) operational taxonomic units (OTU) picking protocols. Further analysis of technical replicates using closed-reference OTU picking showed that the clustering results also have large technical artifacts (Additional file 1). In contrast, the between-replicate similarity on genera- and higher taxonomic levels showed reasonable concordance (Additional file 1). As a result, we implemented the 16S data processing pipeline, which comprised a naive Bayesian classifier from the Ribosomal Database Project [16], and the most recent, full, SILVA database (release 128): we only analyzed taxonomical results using genus- and higher taxonomic levels.

As well as a standard taxonomy binning procedure, all the additional steps have been standardized across the consortium, including downsampling to 10,000 reads with fixed seed to allow for replicability, procedures of transformations, and corrections for covariates, and the thresholds set for bacterial taxa to be included in the analysis (any taxon should be present in more than 10% of the cohort’s samples). This filtering effectively reduces the total number of tests and also makes cross-validation and meta-analysis feasible among all the participating cohorts. 16S data processing is currently being performed in all the cohorts and shows a high level of congruence: the core-measurable microbiome (CMM) [9], defined as the list of bacterial taxa present in more than 10% of the samples in a cohort, is stable across the participating cohorts and shapes around 80% of each cohort’s microbiome composition.

### Genotype data processing

Individual genome-wide genotype data was generated by the different cohort studies using different genotyping platforms and arrays (Table 1). In order to utilize the genome-wide data and remove artifacts resulting from the different platforms, we imputed missing genotypes to extend the resolution on a genome-wide level. We standardized the imputation procedure for each cohort, including the pre-imputation quality control, reference imputation panel, imputation server and software, as well as the post-imputation filtering to include SNPs in the analyses.

Quality control performed prior to imputation was carried out by each cohort independently according to our general recommendations. Imputation was performed on a freely available Michigan server (https://imputationserver.sph.umich.edu/index.html) that uses a two-step approach: phasing with the Eagle v2.3 algorithm, followed by imputation with Minimac [17]. For our consortium, the data was imputed to the Haplotype Reference Consortium (HRC 1.1) reference panels [17]. To allow imputed SNPs in the association studies, we included minor allele frequency filtering (5%), posterior imputation quality (0.4, applied per sample), and variant imputation quality (0.5, applied per SNP). After imputation, each study yielded around 39.1 million SNPs, with 4 to 6 million variants passing post-imputation QC.

### Genome-wide association analysis

Previous microbiome GWAS have used different statistical methods to test association of genetic variants with gut microbiome taxa [9–12], and these might contribute to some of the differences in observed associations. We are therefore developing a uniform analytical pipeline to be implemented by all the studies participating in our consortium; it uses flexible statistical approaches to cope with the non-normality and high dispersion inherent to microbiome data [15]. Several layers of microbiome representations are considered as traits in GWAS: general diversity metrics (alpha- and beta-diversity), series of binomial traits of bacterial presence, and quantitative traits of bacterial relative abundance. At the moment, we are using multiple cohorts for benchmarking, to fine-tune our algorithm and to reduce inter-cohort and technical differences.

### Meta-analyses

Given the substantial increase in sample size (10-fold), as well as our large number of 18 cohorts, we expect to be able to identify individual bacteria and new genomic loci that affect microbiome composition in general. Based on the effect size (0.147 × SD, using a genome-wide threshold of 5e−8) in some 1800 individuals [14], this consortium can theoretically provide 80% power to detect effects larger than 0.045 × SD. Our full pipeline can be found and followed at https://github.com/alexa-kur/miQTL_cookbook. We will also publish summary statistical results from each cohort, as well as the full meta-study results, both on GitHub and as supplementary files in our future publications.

## Conclusions and future directions

The MiBioGen consortium’s large-scale meta-analysis of 18 cohorts drawn from different populations will permit us to explore the genetic architecture of the gut microbiome. In addition to classic association studies, we will adopt more sophisticated approaches to gain a better understanding of the role of the gut microbiome as a mediator between genetic predisposition and human health/disease. For example, we will explore the association of individual risk scores [18] to common diseases, based on published GWAS results and individual microbiome composition.

We will also explore human gene-environment interactions with respect to gut microbiome composition. Such interactions have been observed for the *LCT* non-functional variant and for dairy intake in relation to the abundance of *Bifidobacteria* [10, 19]. Comprehensive studies have explored the independent effects of environmental and genetic forces on the gut microbiome [6, 7, 12–14], and we will investigate a number of gene-environment interactions of interest, including gene-diet, using the combined genetic data and extensive environmental metadata. Certain gene-environment interactions can also be examined in those cohorts that collected stool samples at multiple time points. We appreciate that it will be difficult to determine causality, but we will probably be able to identify a series of environment-gene-microbiome triangles, for instance, those involving age, gender, medication usage, or body mass index. Our results will lead to hypotheses on the links underlying microbiome-related physiological processes. We would therefore encourage any cohorts with an interest in analyzing host-microbiota associations in their own data to join the MiBioGen consortium and to contribute to more overall insights into the intricacies of host genomes’ role in shaping the gut microbiota.

Finally, the additional phenotypes available in each cohort will provide a unique opportunity for quantifying the contribution of the gut microbiome to different phenotypes. For example, GWAS analyses have already been focused on metabolic traits and diseases in different cohorts, and much more cross-checking can be carried out using the EBI GWAS Catalog. The overlap in significant loci will reveal intrinsic relationships between the microbiome, genetics, and diseases, thereby adding to our knowledge of the molecular basis of these pathologies. Recently developed strategies, such as linkage disequilibrium score regression [20] and polygenic risk scores [18], as well as downstream pathway enrichment analyses, will help translate genetic associations into real biological insights into the host-microbiome interaction. Our consortium will thus not only contribute to fundamental knowledge on the gut microbiome but also lead on to clinical and therapeutic efforts in treating diseases.

## Additional files

Additional file 1: Supplementary nformation. (DOCX 361 kb)

Additional file 2: Meta-analysis of human genome-microbiome association studies: the MiBioGen consortium initiative Acknowledgement and funding information. (DOCX 37 kb)

## Abbreviations

CMM: Core-measurable microbiome
EBI: European Bioinformatics Institute
GWAS: Genome-wide association studies
HRC: Haplotype Reference Consortium
QC: Quality control
QTL: Quantitative trait loci
