# Supplementary material for: Meta-analysis of human genome-microbiome association studies: the MiBioGen consortium initiative
Source: Microbiome. 2018 Jun 8;6:101. doi: 10.1186/s40168-018-0479-3 (PMC5992867; doi:10.1186/s40168-018-0479-3)
Supplement: Supplementary file 1 — Supplementary nformation. (DOCX 361 kb) [file 40168_2018_479_MOESM1_ESM.docx]

**Supplementary Information to**

Meta-analysis of human genome-microbiome association studies: the MiBioGen consortium initiative

**Benchmarking of the 16S analytical pipeline**

The different cohorts used different hyper-variable regions of the 16S rRNA gene and different sequencing platforms so it was essential to develop a standardized analytical pipeline to minimize the impact of these differences in the association analyses. The most important step in processing the merged and quality controlled sequences is to generate the taxonomical profiles for all the samples. One method commonly used to generate species-level operational taxonomical units (OTUs, with 97% identity threshold) is clustering sequences *de novo*, and then, based on the taxonomical assignment of each OTU, genus-level consensus and higher taxonomical levels can be calculated. However, because different regions of the 16S rRNA sequencing were amplified by the various platforms, *de novo* clustering could not be performed across different datasets (as there were usually no overlaps between the sequences).However, the profiling could be done by relying on per-sequence classification to a reference database and then generating genus-level and higher taxonomical profiles directly, and independently of any OTU clustering.

In order to reduce artifacts and avoid further complications due to technical differences in our GWAS analyses, we generated a benchmark dataset of ten samples from which cohort(s)?, amplified by V1-V2 primers and MiSeq 2 x 250 bp sequencing, and V4 primers and HiSeq 2 x 100 bp sequencing. For each region/sample, three technical replicates were performed. After standard merging and quality filtering [1], each sample was rarefied to 10,000 reads. Reference-based OTU picking was performed on all samples using the QIIME package [2], using recommended reference databases and 0.97 as the similarity cut-off (i.e., species-level OTUs). Afterwards, genus-level consensus was generated based on the OTUs, by binning all OTUs of the same genus and adding up their relative abundances. At the same time, we used an RDP classifier [3] to generate per-sequence taxonomical assignment and direct genus-level consensus. All taxonomical profiles were then imported in R for analysis. In particular, we performed between-sample analysis using the “Vegan” package in R, where “capscale” package was used to generate the Bray-Curtis dissimilarity based Principal Coordinate Analysis (PCoA), and the “Adonis” test was used to test how much different factors explain the variations in sample differences [4].

The results indicated a relatively large discrepancy in OTU-based profiling between the two variable regions (**Figure S1**). First, the percentage of mapped reads was, on average, significantly higher for the V4 region (94.08 ± 4.16%) than for V1-V2 (89.95 ± 7.13%, Wilcoxon test p = 0.003), which affected various alpha-diversity measures. Second, beta-diversity measurements, which represent between-sample differences, were also primarily driven by the selected region, especially when differences calculated directly from OTUs were used (Adonis test revealed 21.23% of total variation was explained by the different regions, p < 0.001). Even when the OTU-derived genus-level consensus was used, 6.27% of variation was still explained by the regional differences. However, RDP-based classification and the genus-level consensus incorporated all the reads in the final profile and thus removed the effect of the different numbers of reads across samples. This method also reduced the effect of the selected regions (V1-V2 versus V4) to 3.73% percent.

In conclusion, OTU-based analysis is not suitable for comparing and combining sequences generated using different hyper-variable regions, and a more conservative per-sequence classification approach provided better compatibility between the different datasets.

**Figure S1. Overview of OTU-based and direct per-sequence based classification approaches.** A) Percentage of mapped OTUs for different hyper-variable regions. For each sample, two regions-with three technical replicates per region-are shown. B) PCoA analysis using Bray-Curtis dissimilarity calculated from the OTUs identified for each protocol. Samples are distinctly separated by the regions amplified. C) PCoA analysis using Bray-Curtis dissimilarity calculated from OTU-derived genera. Taxonomical profiles from the different amplified regions became more similar, implying a reduction in the impact of the technical artifact. However, 6.27% of the data variation is still explained by differences in amplicon region. D) PCoA analysis using Bray-Curtis dissimilarity and a per-sequence classification using the RDP classifier. This consensus approach further increased the comparability at the genus-level of technical replicates of the same sample sequenced in different regions.

**References**

1 Falony G *et al*. Population-level analysis of gut microbiome variation. Science 352, 560–564 (2016)

2 Caporaso JG *et al.* QIIME allows analysis of high-throughput community sequencing data Nature Methods doi:10.1038/nmeth.f.303 (2010)

3 Wang Q *et al*. Naive Bayesian classifier for rapid assignment of rRNA sequences into the new bacterial taxonomy. Appl Environ Microbiol.  73(16):5261-7 (2007)

4 Anderson MJ. A new method for non-parametric multivariate analysis of variance. Austral Ecology, 26: 32–46 (2001)
