# Supplementary material for: Meta-analysis of human genome-microbiome association studies: the MiBioGen consortium initiative
Source: Microbiome. 2018 Jun 8;6:101. doi: 10.1186/s40168-018-0479-3 (PMC5992867; doi:10.1186/s40168-018-0479-3)
Supplement: Supplementary file 2 — Meta-analysis of human genome-microbiome association studies: the MiBioGen consortium initiative Acknowledgement and funding information. (DOCX 37 kb) [file 40168_2018_479_MOESM2_ESM.docx]

**Meta-analysis of human genome-microbiome association studies: the MiBioGen consortium initiative**

**Acknowledgement and funding information**

**Acknowledgement:**

LLD cohort:

We thank participants and staff of the LifeLines-DEEP cohort for their collaboration. The study was approved by the UMCG review board, ref. M12.113965. We thank Jackie Dekens, Mathieu Platteel, Astrid Maatman and Jody Arends for management and technical support.

METSIM cohort:

We thank all METSIM study participants for their collaboration. The study was approved by the Ethics Committee of the University of Kuopio and was in accordance with the Helsinki Declaration.

TwinsUK:

We thank participants and staff of the TwinsUK cohort for their collaboration.  Ethics approval for the TwinsUK study was given by the NRES Committee London - Westminster (REC 520  Reference No. : EC04/015), with all participants providing written consent.

SHIP cohort:

SHIP is part of the Research Network Community Medicine of the University Medicine Greifswald, Germany, which is supported by the German Federal State of Mecklenburg – West Pomerania.

GEM cohort:

We thank the members of the GEM Global Project Office: Cristina Bravi, Deema Couchman, Nisha Ganeswaren, Alexandra Keludjian, Kevin Ow, Rachel Caplan, Melissa Greaves, Amy Craig-Neil, Ana Olteanu, Nellie Allam, Amanda Garrioch, Denis Ng, Venus Onay and Isabelle Yeadon for administrative support. We thank Dennis Cvitkovitch for his helpful scientific discussion of the project. The CCC IBD GEM Project research team is of: Maria Abreu, Paul Beck, Charles Bernstein, Kenneth Croitoru, Leo Dieleman, Brian Feagan, Anne Griffiths, David Guttman, Kevan Jacobson, Gilaad Kaplan, Denis O. Krause*, Karen Madsen, John Marshall, Paul Moayyedi, Mark Ropeleski, Ernest Seidman, Mark Silverberg, Scott Snapper, Andy Stadnyk, Hilary Steinhart, Michael Surette, Dan Turner, Tom Walters, Bruce Vallance, Guy Aumais, Alain Bitton, Maria Cino, Jeff Critch, Lee Denson, Colette Deslandres, Wael El-Matary, Hans Herfarth, Peter Higgins, Hien Huynh, Jeff Hyams, David Mack, Jerry McGrath, Anthony Otley, and Remo Panancionne. (* deceased).

Rotterdam Study:

The generation and management of GWAS genotype data for the Rotterdam Study (RS I, RS II, RS III) was executed by the Human Genotyping Facility of the Genetic Laboratory of the Department of Internal Medicine, Erasmus MC, Rotterdam, The Netherlands. We thank Pascal Arp, Mila Jhamai, Marijn Verkerk, Lizbeth Herrera, dr. Marjolein Peters and dr. Carolina Medina-Gomez for their help in creating the GWAS database, and dr. Karol Estrada, dr. Yurii Aulchenko and dr. Carolina Medina-Gomez for the creation and analysis of imputed data. The authors are very grateful to the study participants, the staff from the Rotterdam Study (particularly L. Buist and J.H. van den Boogert) and the participating general practitioners and pharmacists.

Generation R Study:

The Generation R Study is conducted by the Erasmus Medical Center in close collaboration with the School of Law and Faculty of Social Sciences of the Erasmus University Rotterdam, the Municipal Health Service Rotterdam area, Rotterdam, the Rotterdam Homecare Foundation, Rotterdam and the Stichting Trombosedienst & Artsenlaboratorium Rijnmond [STAR-MDC], Rotterdam. We gratefully acknowledge the contribution of children and parents, general practitioners, hospitals, midwives and pharmacies in Rotterdam. The study protocol was approved by the Medical Ethical Committee of the Erasmus MC, Rotterdam. Written informed consent was obtained for all participants. The generation and management of GWAS genotype data for the Generation R Study was done at the Genetic Laboratory of the Department of Internal Medicine, Erasmus MC, the Netherlands. We would like to thank Karol Estrada, Dr. Tobias A. Knoch, Anis Abuseiris, Luc V. de Zeeuw, and Rob de Graaf, for their help in creating GRIMP, BigGRID, MediGRID, and Services@MediGRID/D-Grid, [funded by the German Bundesministerium fuer Forschung und Technology; grants 01 AK 803 A-H, 01 IG 07015 G] for access to their grid computing resources. We thank Pascal Arp, Mila Jhamai, Marijn Verkerk, Manoushka Ganesh, Lizbeth Herrera and Marjolein Peters for their help in creating, managing and QC of the GWAS database.

KSCS cohort:

We thank participants and staff of the Kangbuk Samsung Cohort Study for their collaboration, especially Yoosoo Chang, Seungho Ryu, and Hocheol Shin. The study was approved by the EUMC review board 2014-06-024 and KBSMC review board 2013-01-245. The computing resources was supported by Global Science experimental Data hub Center (GSDC) Project and Korea Research Environment Open NETwork (KREONET) in Korea Institute of Science and Technology Information (KISTI).

The PopCol study was approved by Karolinska Institutet’s ethics committee, Stockholm, Sweden (dnr 394/01).

**Funding information**:

LLD cohort:

This project was funded by the Netherlands Heart Foundation (IN-CONTROL CVON grant 2012-03 to A.Z. and J.F.), by the Top Institute Food and Nutrition, Wageningen, the Netherlands (TiFN GH001to C.W.), by the Netherlands Organization for Scientific Research (NWO) (NWO-VIDI 864.13.013 to J.F., NWO-VIDI 016.178.056 to A.Z., NWO-VIDI 917.14.374 to L.F., and NWO Spinoza Prize SPI 92-266 to C.W.), and by the European Research Council (ERC) (FP7/2007-2013/ERC Advanced Grant agreement 2012-322698 to C.W., ERC Starting Grant 715772 to A.Z and ERC Starting Grant 637640 to L.F.). A.Z. also holds a Rosalind Franklin Fellowship from the University of Groningen

METSIM cohort:

This study was supported by Academy of Finland ([www.aka.fi](http://www.aka.fi/)) grants 77299 and 124243 to ML, by the Juselius Foundation to ML, by the Finnish Heart Foundation ([www.sydantutkimussaatio.fi/en/grants](http://www.sydantutkimussaatio.fi/en/grants)) to ML, by the Finnish Diabetes Foundation ([www.diabetestutkimus.fi/](http://www.diabetestutkimus.fi/)) to ML, by the National Institutes of Health (NIH) grants HL028481, HL30568, and DK094311 to AJL, DK062370 to MB, DK072193 to KM and by the Estonian Research Council Starting Grant PUT1371 to E.O.

TwinsUK:

The TwinsUK microbiota project was funded the National Institutes of Health (NIH) RO1 DK093595, DP2 OD007444. TwinsUK received funding from the Wellcome Trust; European Community’s Seventh Framework Programme (FP7/ 2007-2013), the National Institute for Health Research (NIHR)-funded BioResource, Clinical Research Facility and Biomedical Research Centre based at Guy’s and St Thomas’ NHS Foundation Trust in partnership with King’s College London. CJS is funded under a grant from the Chronic Disease Research Foundation (CDRF). TS is NIHR Senior investigator.

NGRC Cohort:

We thank participants and scientists of NeuroGenetics Research Consortium. The project was funded by the National Institute of Neurologic Disease and Stroke (NS036960 to HP).

Popgen/Focus:

This study was supported by the German Ministry of Education and Research (BMBF) program e:Med sysINFLAME (http://www. gesundheitsforschung-bmbf.de/de/5111.php, no.: 01ZX1306A), the Deutsche Forschungsgemeinschaft (DFG) Cluster of Excellence ‘Inflammation at Interfaces’ (http://www.inflammation-at-interfaces.de, no.: EXC306 and EXC306/2) and Collaborative Research Center 1182 ‘Origin and Function of Metaorganisms’ (<http://www.metaorganism-research.com>, DFG grant BO 848/17–1). The POPGEN 2.0 Network was supported also by the German Ministry of Education and Research (01EY1103).

This study was supported by grants from Crohn's and Colitis Canada, Canadian Institutes of Health Research (CIHR) Grant #CMF108031 and the Helmsley Charitable Trust. Williams Turpin is a recipient of a Postdoctoral Fellowship Research Award from the CIHR Fellowship/ Canadian Association of Gastroenterology (CAG)/ Ferring Pharmaceuticals Inc. and a fellowship from the Department of Medicine, Mount Sinai Hospital, Toronto. Mark Silverberg is supported in part by the Gale and Graham Wright Chair in Digestive Diseases. Osvaldo Espin-Garcia is a fellow trainee of CIHR STAGE (Strategic Training for Advanced Genetic Epidemiology) - CIHR Training Grant in Genetic Epidemiology and Statistical Genetics. CIHR Training Grant GET-101831

NeuroIMAGE+COMPULS:

This project has received funding from the European Union’s Horizon 2020 research and innovation programme under the Marie Sklodowska-Curie grant agreement No 643051.

Rotterdam Study:

The Rotterdam Study is funded by Erasmus Medical Center and Erasmus University, Rotterdam, Netherlands Organization for the Health Research and Development (ZonMw), the Research Institute for Diseases in the Elderly (RIDE), the Ministry of Education, Culture and Science, the Ministry for Health, Welfare and Sports, the European Commission (DG XII), and the Municipality of Rotterdam. The GWAS datasets are supported by the Netherlands Organisation of Scientific Research NWO Investments (nr. 175.010.2005.011, 911-03-012), the Genetic Laboratory of the Department of Internal Medicine, Erasmus MC, the Research Institute for Diseases in the Elderly (014-93-015; RIDE2), the Netherlands Genomics Initiative (NGI)/Netherlands Organisation for Scientific Research (NWO) Netherlands Consortium for Healthy Aging (NCHA), project nr. 050-060-810.

Generation R Study:

The general design of Generation R Study is made possible by ﬁnancial support from the Erasmus Medical Center, Rotterdam, the Erasmus University Rotterdam, the Netherlands Organization for Health Research and Development (ZonMw), the Netherlands Organisation for Scientific Research (NWO), the Ministry of Health, Welfare and Sport and the Ministry of Youth and Families.

KSCS cohort:

This project was funded by the Korea Health Industry Development Institute (KHIDI) of the Ministry of Health & Welfare (KHIDI grant HI14C0072), and by the National Research Foundation of Korea (NRF) (NRF-2014R1A2A2A04006291 and NRF-2016R1A6A3A11932719).

Personal Nutrition Project:

E.S. is supported by the Crown Human Genome Center; the Else Kroener Fresenius Foundation; Donald L. Schwarz, Sherman Oaks, CA; Jack N. Halpern, New York, NY; Leesa Steinberg, Canada; and grants funded by the European Research Council and the Israel Science Foundation.

PopCol:

Genetic and microbiota analyses in PopCol are supported by funding from the Swedish Research Council (VR) to MDA and the European Union Seventh Framework Programme (FP7/2007–2013, ESGI) to MDA and AF.

FGFP cohort:

The FGFP cohort organized by Jeroen Raes lab was funded with support of the Flemish government (IWT130359), the Research Fund–Flanders (FWO project number G.0924.09 and G095516N), the King Baudouin Foundation (2012-J80000-004), , FP7 METACARDIS HEALTH-F4-2012-305312, VIB, the Rega institute for Medical Research, and KU Leuven. JW is supported by a Research Fund–Flanders (FWO) postdoc grant, and Key Research Program of Chinese Academy of Science Grant No. KFZD-SW-219, “China Microbiome Initiative”. NJT is a Wellcome Trust Investigator (202802/Z/16/Z), a programme lead in the MRC Integrative Epidemiology Unit (MC_UU_12013/3) and works within the University of Bristol NIHR Biomedical Research Centre (BRC). NJT and DAH work in the Medical Research Council Integrative Epidemiology Unit (IEU) at the University of Bristol which is supported by the Medical Research Council (MC_UU_12013/1-9).

We thank Jackie Senior for editing the manuscript.
